# Supplementary material for: Cryo-EM structure of human Pol κ bound to DNA and mono-ubiquitylated PCNA
Source: Nat Commun. 2021 Oct 19;12:6095. doi: 10.1038/s41467-021-26251-6 (PMC8526622; doi:10.1038/s41467-021-26251-6)
Supplement: Supplementary file 1 — Supplementary Information [file 41467_2021_26251_MOESM1_ESM.pdf]

Supplementary Information

Cryo-EM structure of human Pol κ bound to DNA and mono-ubiquitylated PCNA

Lancey, et al.

Includes:

Supplementary Figures 1-13
Supplementary Methods
Description of Supplementary Movies 1-4
Supplementary Tables 1-2

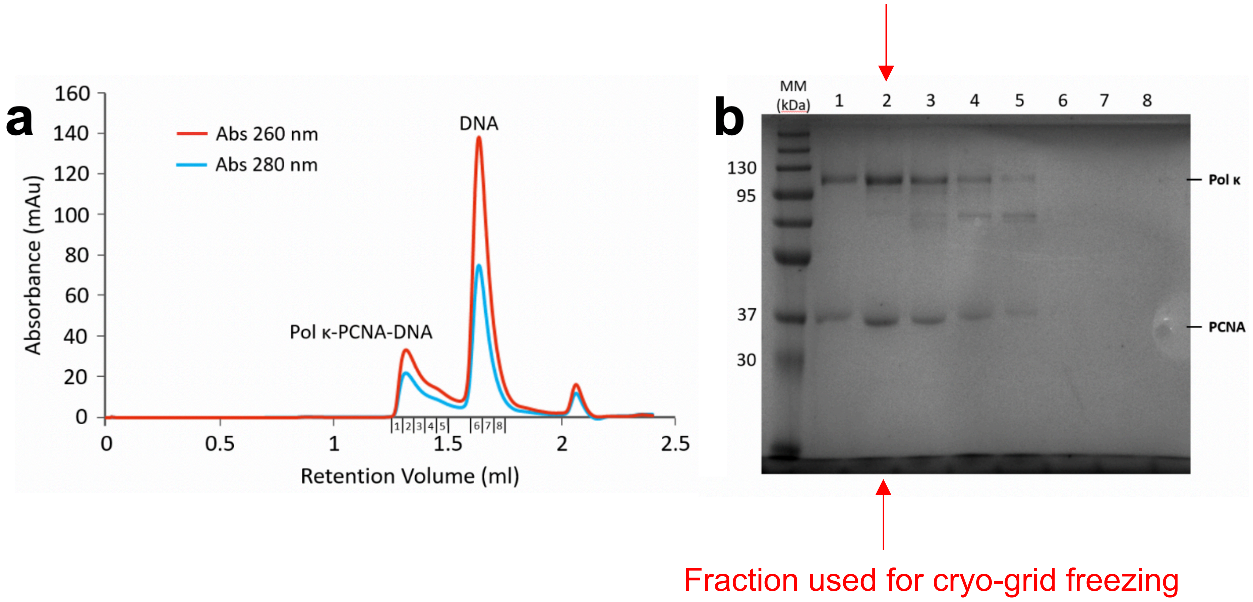

Supplementary Figure 1. Sample separation of the Pol κ–DNA–PCNA complex. **a)** Gel filtration chromatography of the reconstituted Pol κ–DNA–PCNA complex. **b)** The numbered peak fractions in a) were analyzed by SDS-PAGE (lanes 1–8). Proteins corresponding to the bands are labeled on the right. Molecular weight standards are shown on the left.

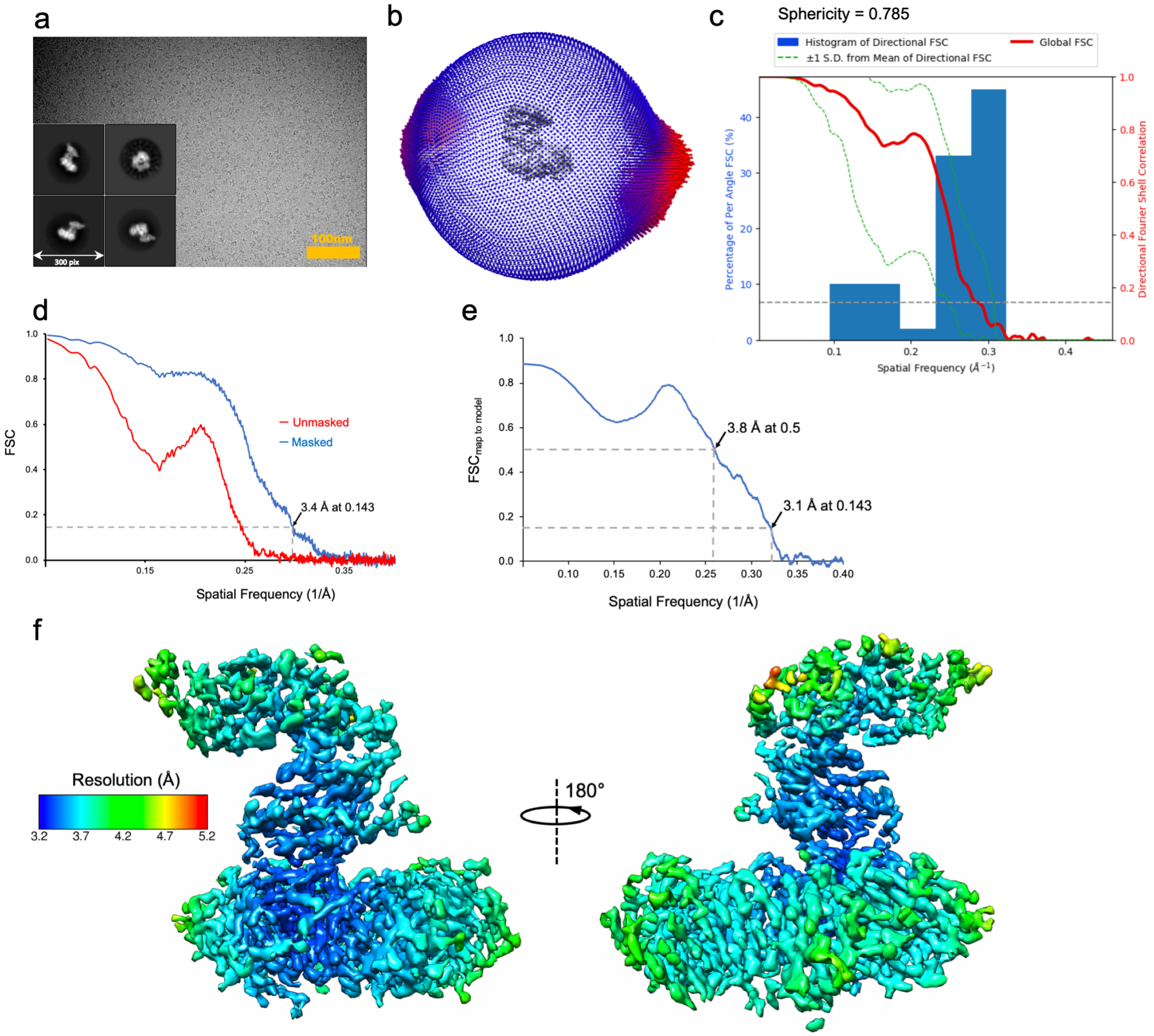

Supplementary Figure 2. Cryo-EM of the Pol κ–DNA–wtPCNA complex. **a)** Representative electron micrograph (aligned sum) acquired using a Gatan K3 direct electron detector in super resolution mode, and representative 2D class averages. A total of 11114 micrographs were acquired. **b)** Angular distribution of projections. **c)** Map anisotropy analysis computed by 3DFSC[1]. The grey dotted line corresponds to FSC = 0.143. **d)** Gold-standard Fourier shell correlation, and resolution estimation using the 0.143 criterion. **e)** Map-to-model Fourier shell correlation. **f)** Two views of the cryo-EM map colored by local resolution.

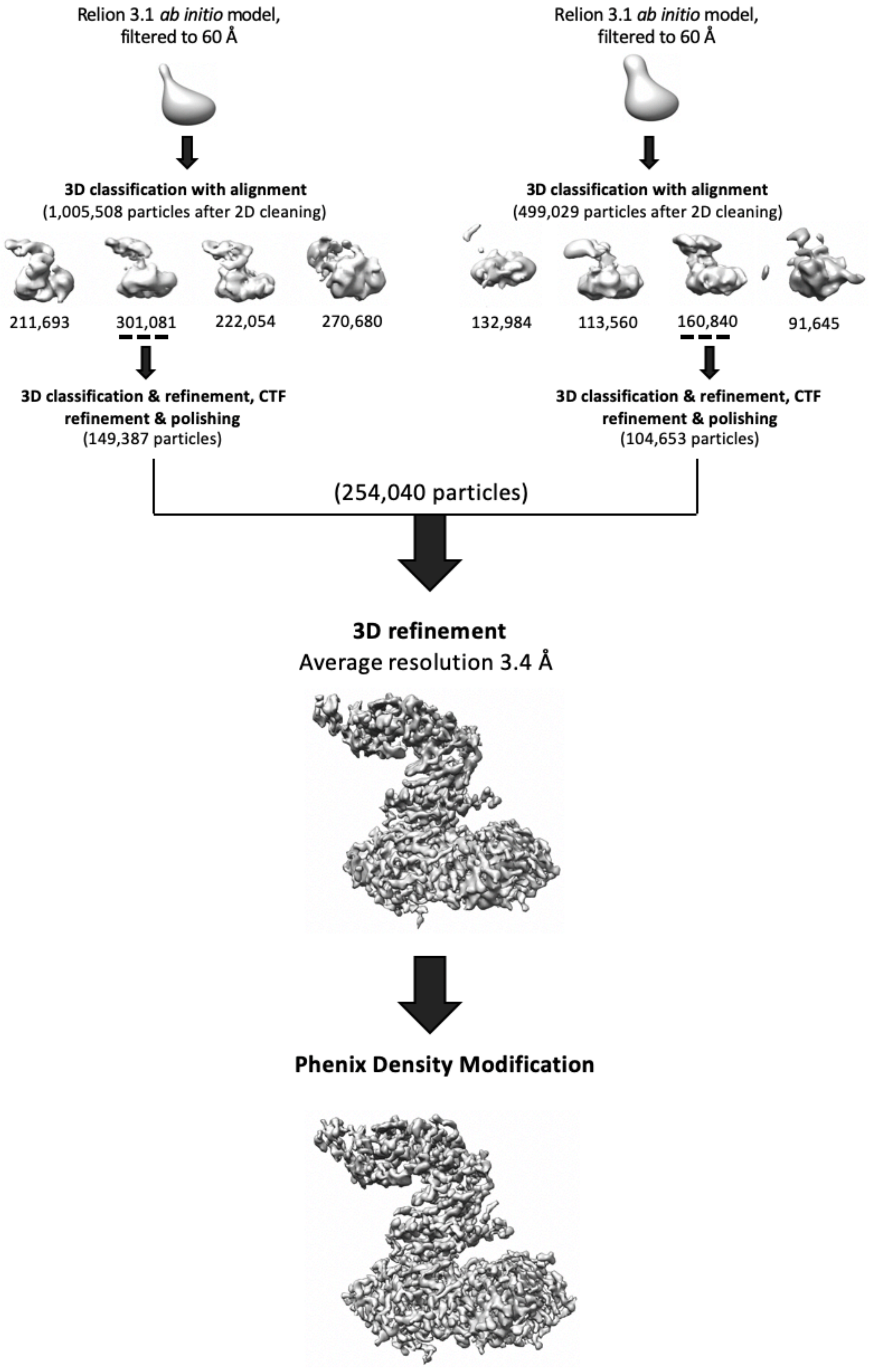

Supplementary Figure 3. Overview of image processing of the Pol κ–DNA–wtPCNA complex.

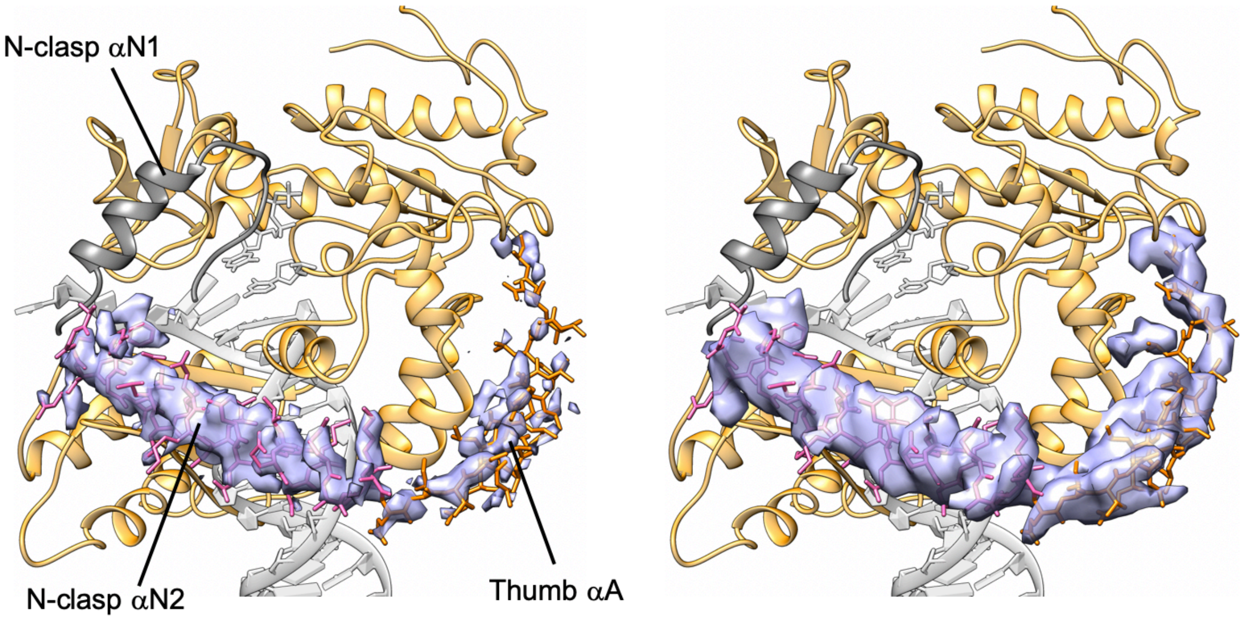

Supplementary Figure 4. Cryo-EM of the Pol κ–DNA–wtPCNA complex**.** *Left:* Cryo-EM density around the N-clasp αN2 and thumb helix αA showing that αA is poorly defined, suggesting that it is dynamic. *Right:* helix αA becomes fully visible after blurring the map with a B factor of 20 Å^2. Density for N-clasp αN1 is not observed in the map.

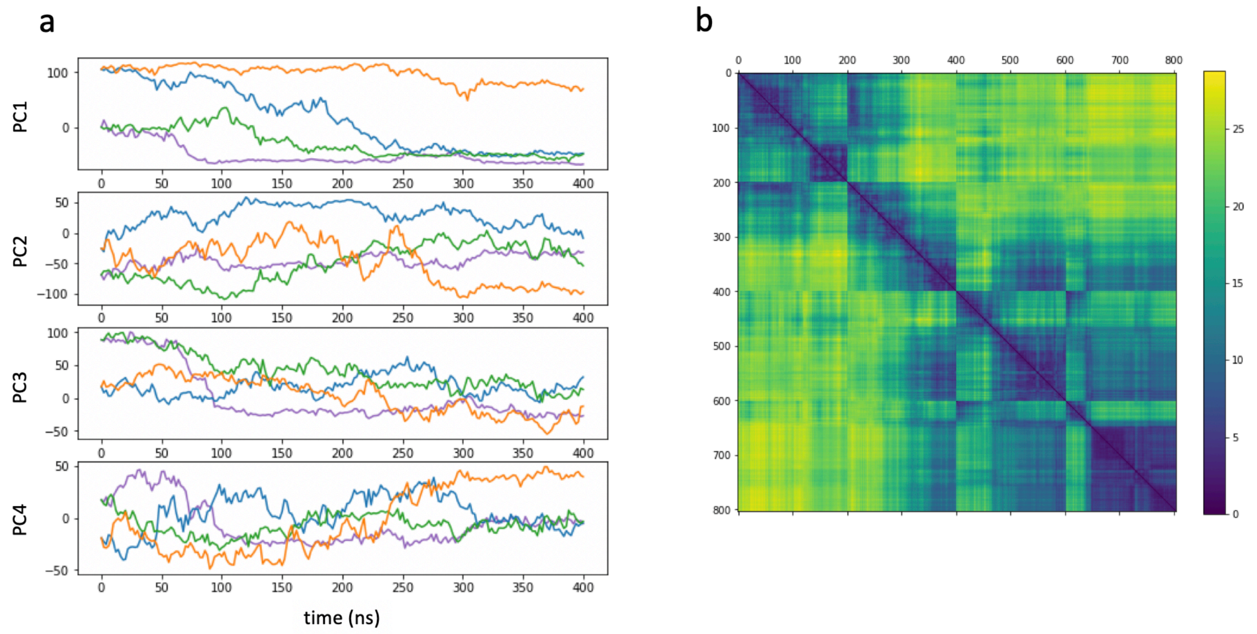

Supplementary Figure 5. a) Time evolution of the projections of the four MD trajectories onto the first 4 principal components (PC). Trace color code is: Orange, apo1; Blue, apo1b; Green, apo2; Purple, apo2b. **b)** Cross RMSD in Å of all frames in all 4 trajectories. The 800 structures represent the 200 frames of each of the 4 trajectories appended one after the other. The order of the trajectories is apo1, apo1b, apo2, apo2b. The figure shows that, although no trajectory samples the full configurational space, because trajectories mainly resemble to themselves, some trajectories sample conformations similar to others. In particular, apo1b (structures from 200 to 400) samples conformations that are closer to the ones sampled by trajectories starting from apo2 (structures from 400 to 800). This is seen from the low RMSD values between structures in the range of 300-400 with structures in the range from 400-800.

a

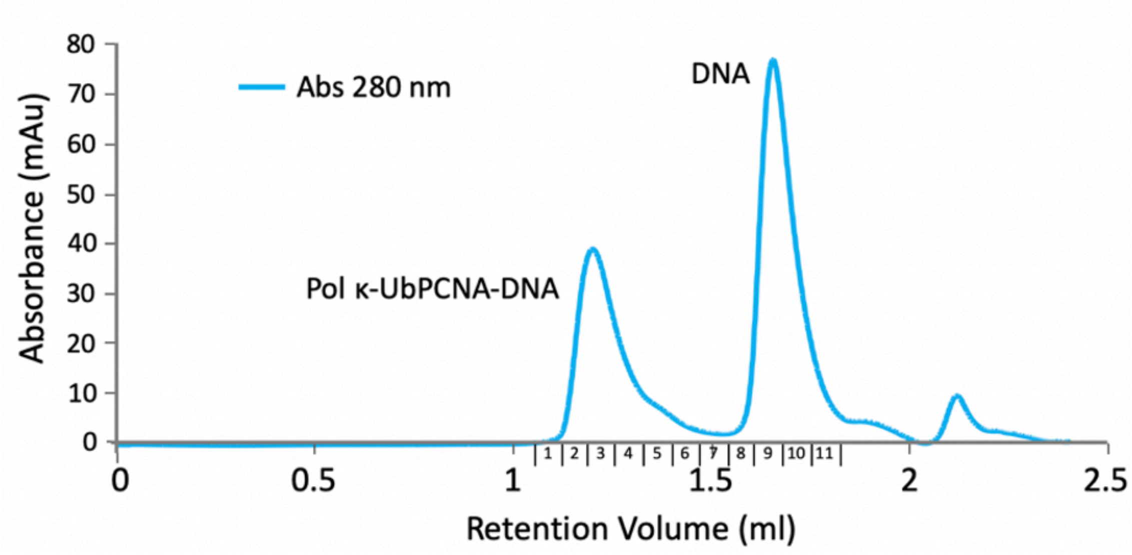

b

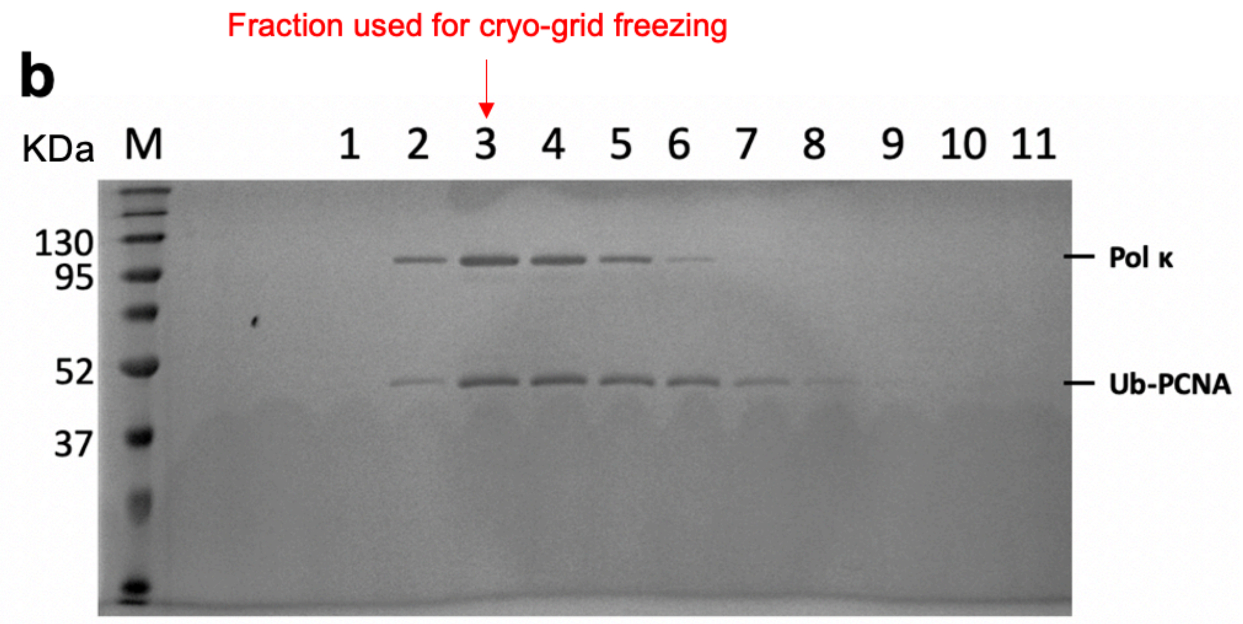

Supplementary Figure 6. Sample separation of the Pol κ–DNA–UbPCNA complex. **a)** Gel filtration chromatography of the reconstituted Pol κ–DNA–UbPCNA complex. **b)** The numbered peak fractions in a) were analyzed by SDS-PAGE (lanes 1–8). Proteins corresponding to the bands are labeled on the right. Molecular weight standards are shown on the left.

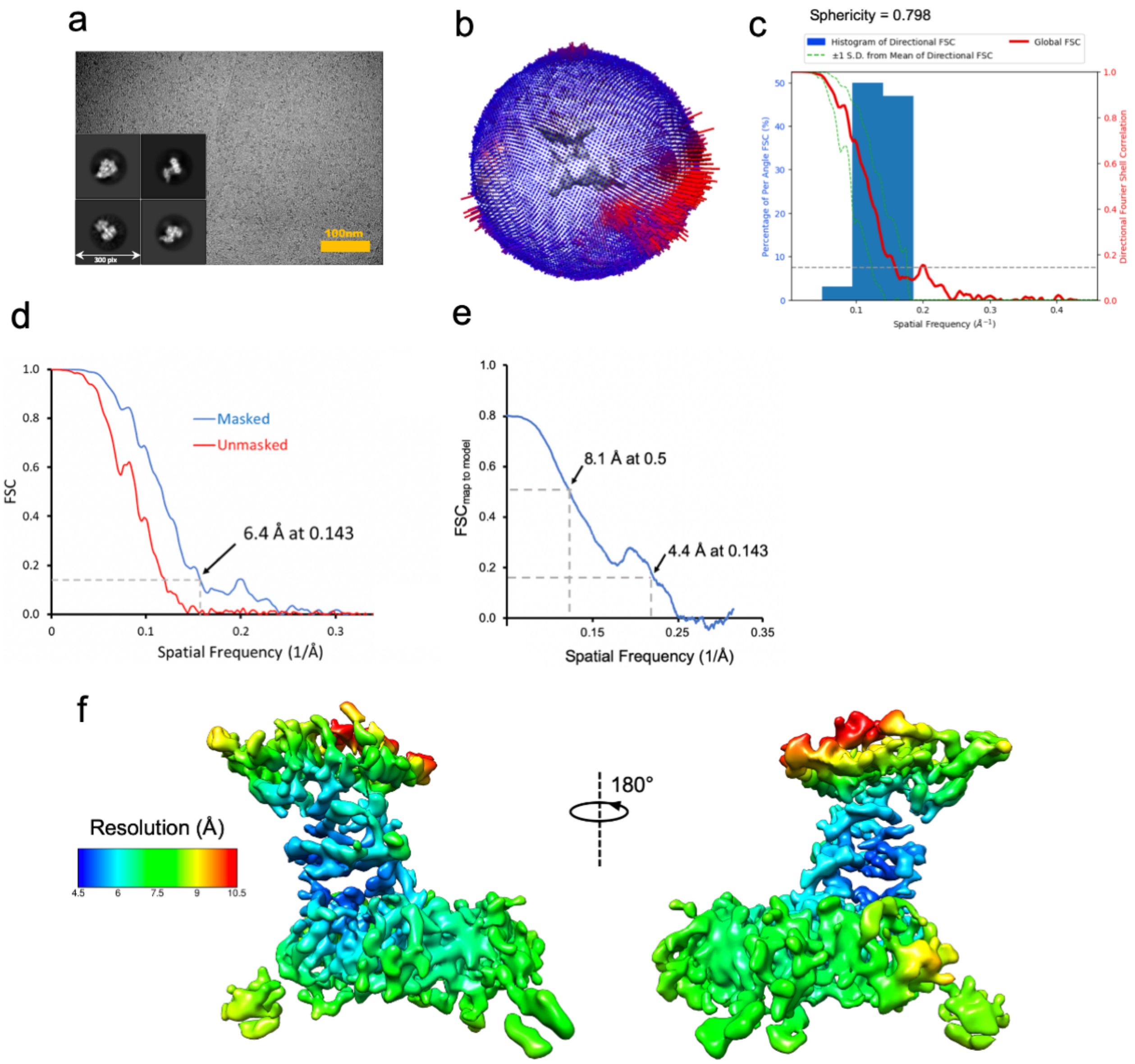

Supplementary Figure 7. Cryo-EM of the Pol κ–DNA–UbPCNA complex. **a)** Representative electron micrograph (aligned sum) acquired using a Gatan K3 direct electron detector in super resolution mode, and representative 2D class averages. A total of 3389 micrographs were acquired. **b)** Angular distribution of projections of the 6.1 Å reconstruction. **c)** Map anisotropy analysis computed by 3DFSC[1]. The grey dotted line corresponds to FSC = 0.143. **d)** Gold-standard Fourier shell correlation, and resolution estimation using the 0.143 criterion. **e)** Map-to-model Fourier shell correlation. **f)** Two views of the cryo-EM map colored by local resolution.

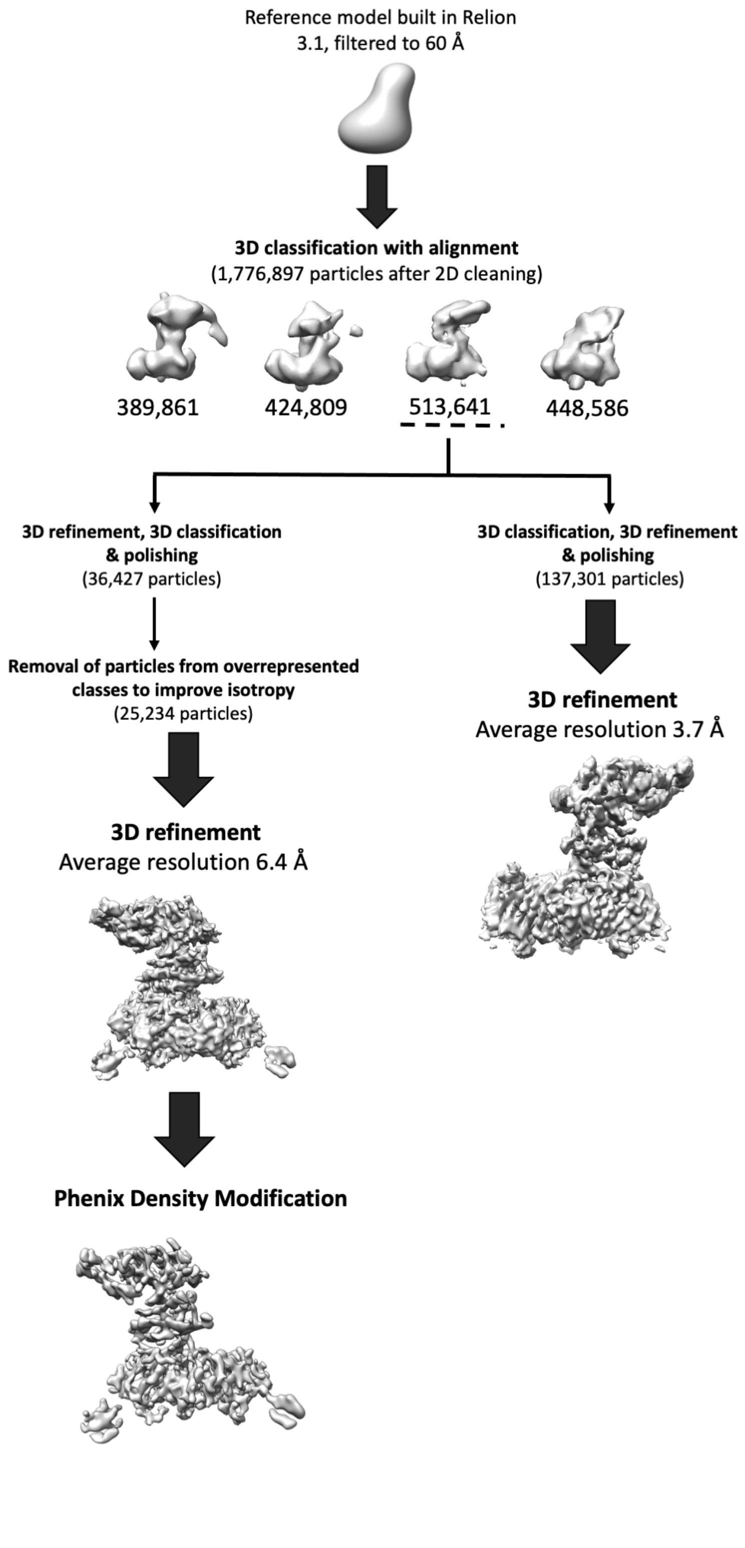

Supplementary Figure 8. Overview of image processing of the Pol κ–DNA–UbPCNA complex.

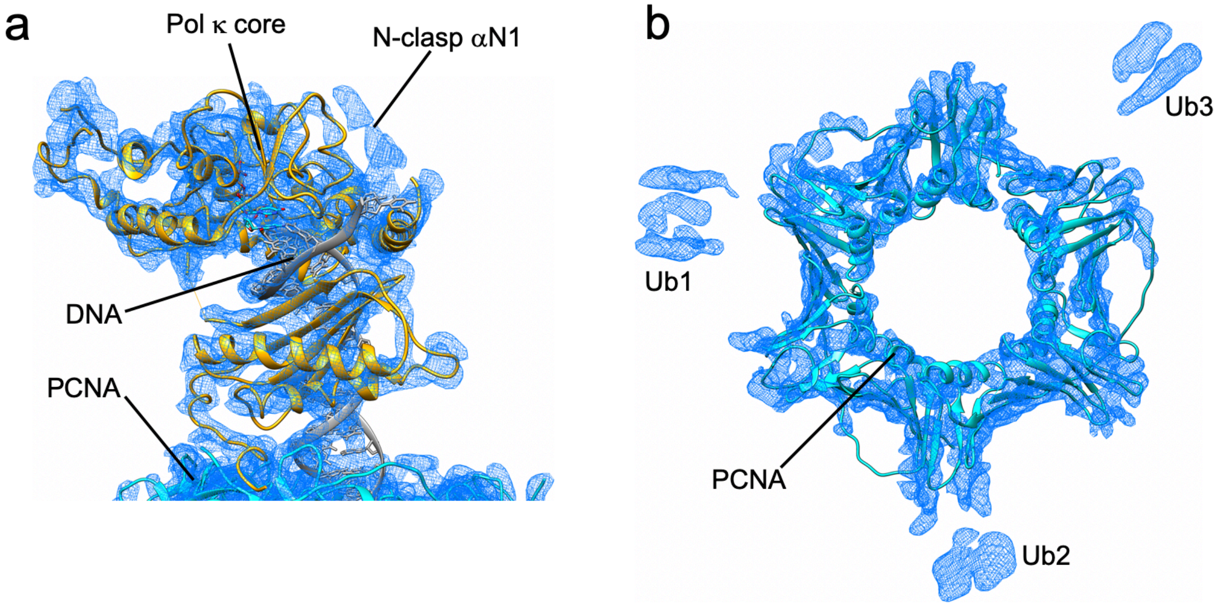

Supplementary Figure 9. Cryo-EM map and model of the Pol κ–DNA–UbPCNA complex**. a)** Cryo-EM density around Pol κ core. **b)** Cryo-EM density around PCNA.

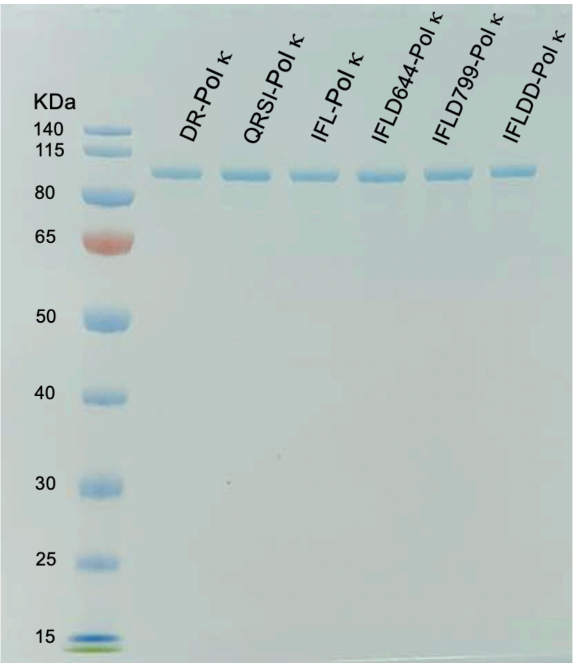

Supplementary Figure 10. SDS-PAGE gel showing the purified full-length human Pol κ mutants.

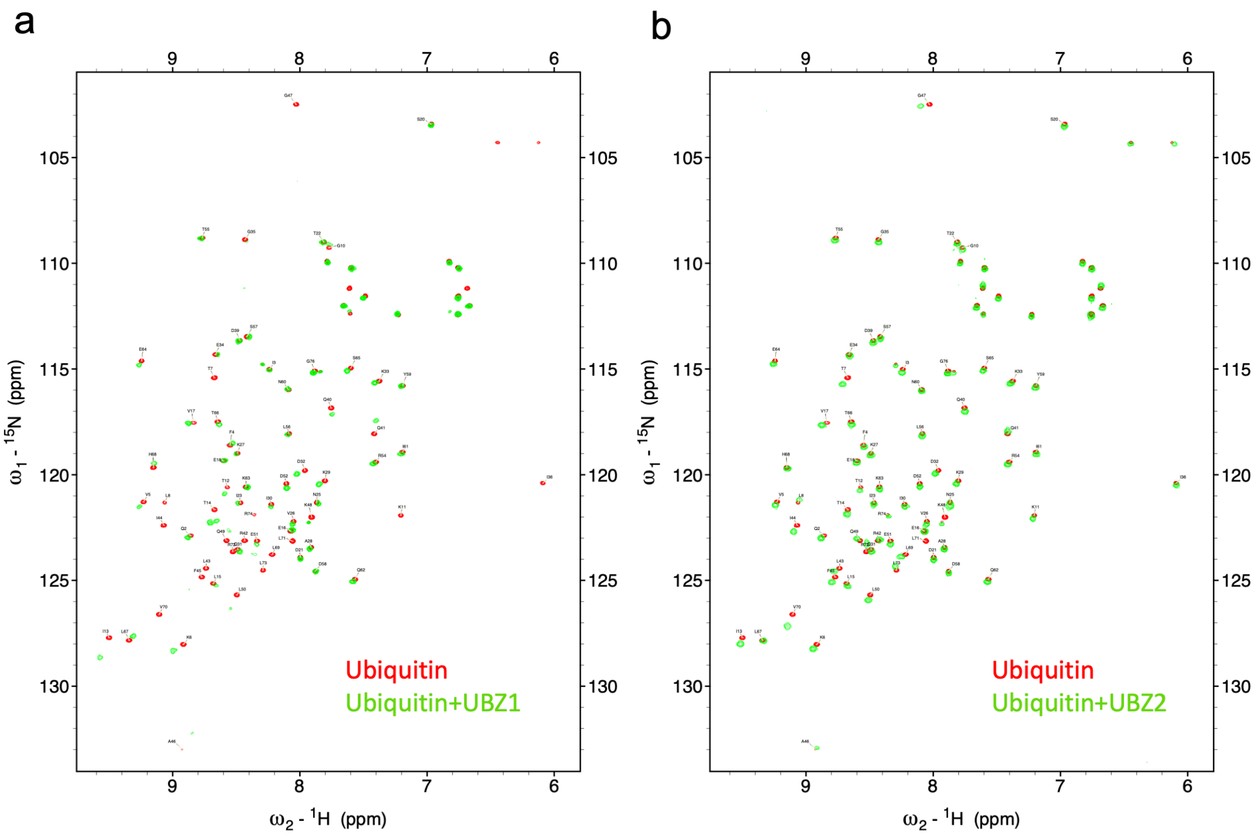

Supplementary Figure 11. a) ^{15}N-^{1}H HSQC spectra of ^{15}N-labelled ubiquitin in the absence (red) or presence (green) of a 10-fold excess of unlabelled Pol κ UBZ1. **b)** ^{15}N-^{1}H HSQC spectra of ^{15}N-labelled ubiquitin in the absence (red) or the presence (green) of a 10-fold excess of unlabelled Pol κ UBZ2.

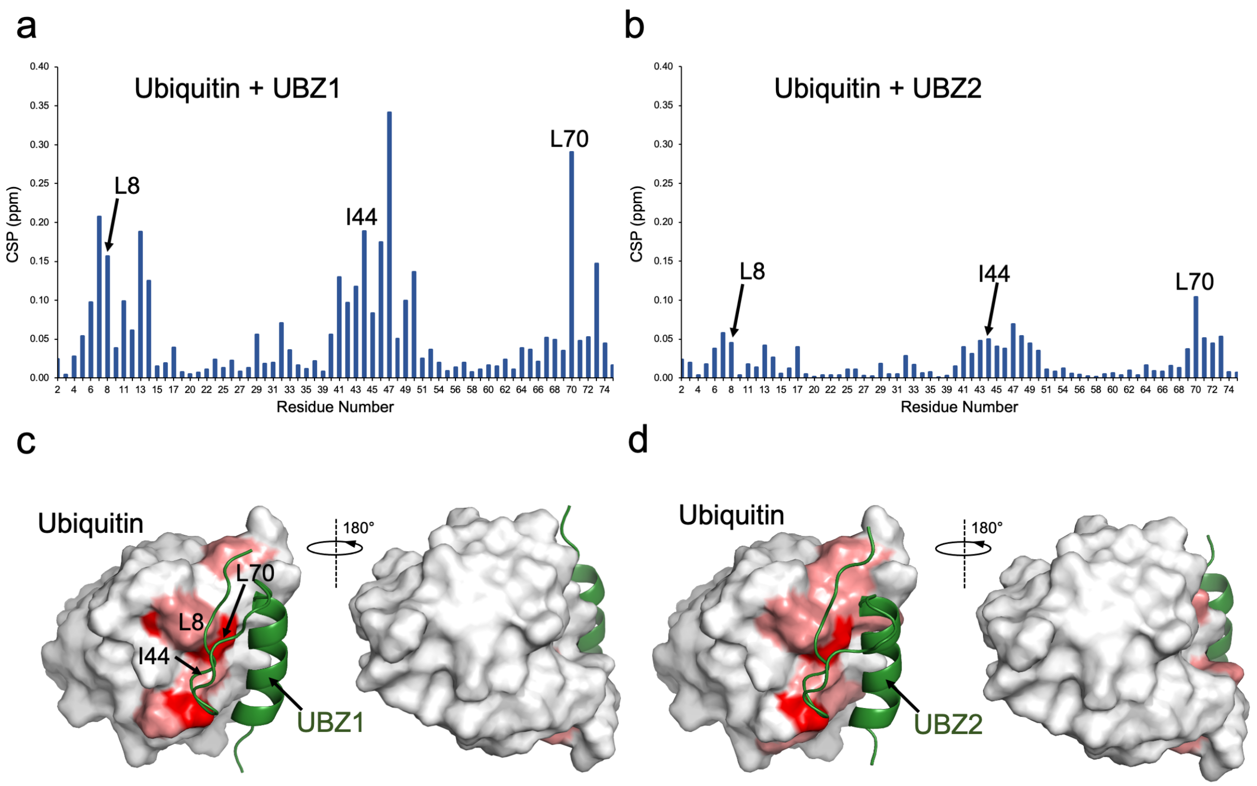

Supplementary Figure 12. (a and b) Per-residue chemical shift differences between bound and free ^{15}N-labeled ubiquitin upon addition of unlabelled Pol κ UBZ1 or UBZ2. (**c** and **d**) Binding-induced chemical shift perturbations for the backbone amide groups (shown in panels a and b) mapped onto the ubiquitin structure. Residues with a chemical shift difference larger than the mean plus 1 and 2 standard deviations are colored pink and red, respectively. The homology models for ubiquitin bound to UBZ1 or UBZ2 were built as shown in main text Figure 5.

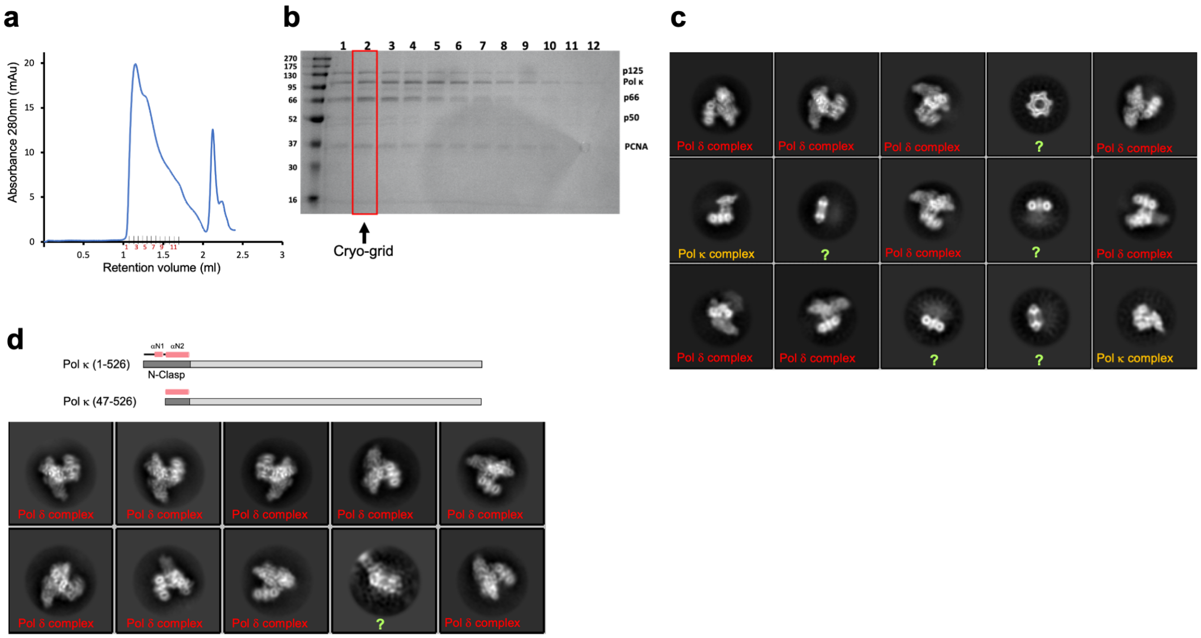

Supplementary Figure 13. *Attempts to reconstitute a Pol κ–Pol δ–PCNA–DNA toolbelt and visualize it by cryo-EM*. **a)** Gel filtration chromatography of a mixture containing Pol κ, Pol δ, PCNA, P/T DNA and dTTP. **b)** SDS-PAGE of the eluted fractions. The fraction boxed in red was vitrified and imaged by cryo-EM. **c)** 2D class averages of the sample eluted by gel filtration, showing the presence of either Pol δ–DNA–PCNA complex or Pol κ–DNA–PCNA complex. Some 2D classes (labeled with a question mark) show PCNA surrounded by a fuzzy density that could not be clearly assigned. **d)** In an attempt to capture a transient state of the complex with both Pol δ and Pol κ bound to PCNA but with Pol κ disengaged from P/T DNA, a Pol κ variant lacking 46 residues at the N-terminus was expressed and purified. Such N-terminal deletion has been previously shown to decrease the DNA binding affinity of Pol κ by 4-fold[2]. This Pol κ mutant was mixed with Pol δ, PCNA, P/T DNA and dTTP, and the mixture was vitrified and imaged by cryo-EM. 2D class averages only showed the presence of Pol δ complex, with no indication of Pol κ bound.

Supplementary Methods

Pol δ preparation

Human Pol δ was cloned and expressed as described previously[3]. Briefly, all four Pol δ subunits encoding p125 (accession no. NP02682), p50 (accession no. NP006221), p66 (accession no. NP006582) and p12 (accession no. NP066996) were amplified and cloned to make a single MultiBac™ expression plasmid. Bacmid DNA was produced by transforming a single recombinant transfer vector encoding all four Pol δ subunits into DH10MultiBac™ cells. To prepare the baculovirus, bacmid DNA containing all four

subunits was transfected into Sf9 cells using FuGENE® HD (Promega) according to the manufacturer's instructions. This baculovirus prep was then amplified twice to obtain a higher titer virus (P3 virus). The expression of Pol δ then proceeded by transfecting a 4 L Sf9 suspension culture at a density of 2×10^6 cells/mL with the P3 virus for 66-72 hrs. Pol δ clear lysate was loaded on a HisTrap and eluted with low salt, followed by ion-exchange chromatography on a Mono Q column, and finally size exclusion chromatography on HiLoad 16/600 Superdex 200 pg. Protein fractions were pooled, flash frozen and stored at −80°C.

$^{47\text{-}526}$Pol κ preparation

The First 46 amino acids of Pol κ were deleted by PCR to generate $^{47\text{-}526}$Pol κ mutant and confirmed by sequencing. The $^{47\text{-}526}$Pol κ mutant was expressed and purified as described in Materials and Methods for Pol κ and its mutants' purifications. Briefly, BL21 (DE3) cells overexpressing the mutant were harvested after induction with IPTG and 19 hrs of incubation at 16 ^{o}C. Cells were re-suspended in lysis buffer and lysed by lysozyme followed by sonication. Cell debris was then removed by centrifugation and loaded directly onto the HisTrap HP 5ml column. Bound protein was eluted with an imidazole gradient. The fractions containing the $^{47\text{-}526}$Pol κ mutant was pooled, and SUMO protease was added to cleave the SUMO tag and generate Pol κ in the native form and dialyzed overnight in the dialysis buffer as described in the Materials and Methods section. The dialyzed protein was then loaded again onto the HisTrap HP 5ml column and the native protein was collected in the flow-through fractions. Fractions that contained $^{47\text{-}526}$Pol κ mutant were pooled, concentrated and loaded onto HiLoad 16/600 Superdex 200 pg (GE Healthcare) equilibrated with storage buffer [50 mM Tris (pH 7.5), 300 mM NaCl and 1 mM DTT]. Fractions containing $^{47\text{-}526}$Pol κ mutant were checked for purity, concentrated, flash frozen and stored at -80 °C.

Cryo-EM methods for Supplementary Figure 13

For the Pol κ-Pol δ toolbelt, a 25 µl inject containing 3 µM P/T DNA, 3 µM Pol δ, 3 µM Pol κ, 3 µM PCNA trimer and 20 µM TTP was loaded onto a Superdex 200 increase 3.2/300 column (GE Life Sciences), equilibrated with a buffer comprising (25 mM HEPES (pH

7.5), 100 mM potassium acetate, 10 mM calcium chloride, 0.02% NP-40, 0.5 mM TCEP). 3 µl of a fraction corresponding to the first peak (Supplementary Figure 13, fraction and lane 2) was used. The cryo grid preparation and cryo-EM data collection were done in the same way with the same parameters as for the other complexes, as described in the main methods section.

Supplementary Movie 1.
Apo1 MD trajectory of Pol κ–PCNA complex. Colour code: Pol κ core in bright orange; Pol κ PAD in green; PAD C-terminus in red; PCNA in sky blue.
Supplementary Movie 2.
Apo1b MD trajectory of Pol κ–PCNA complex. Colour code: Pol κ core in bright orange; Pol κ PAD in green; PAD C-terminus in red; PCNA in sky blue.
Supplementary Movie 3.
Apo2 MD trajectory of Pol κ–PCNA complex. Colour code: Pol κ core in bright orange; Pol κ PAD in green; PAD C-terminus in red; PCNA in sky blue.
Supplementary Movie 4.
Apo2b MD trajectory of Pol κ–PCNA complex. Colour code: Pol κ core in bright orange; Pol κ PAD in green; PAD C-terminus in red; PCNA in sky blue.

Supplementary Table 1. Cryo-EM data collection, refinement and validation statistics

	Polκ-DNA-dTTP-wtPCNA (EMD-12601) (PDB 7NV0)	Polκ-DNA-dTTP-UbPCNA (visible Ub) (EMD-12602) (PDB 7NV1)
**Data collection and processing**		
Magnification	81,000	81,000
Voltage (kV)	300	300
Electron exposure (e–/Å^2)	47	47
Defocus range (µm)	-0.8 to -2.0	-0.8 to -2.0
Pixel size (Å)	1.086	1.086
Symmetry imposed	C1	C1
Initial particle images (no.)	12,741,853	5,275,717
Final particle images (no.)	254,040	25,234
Map resolution (Å)	3.4	6.4
FSC threshold	0.143	0.143
Map resolution range (Å)	3.31 – 5.05	4.82 – 13.1
**Refinement**		
Initial model used (PDB code)	2OH2, 6TNY	2OH2, 6TNY
Model resolution (Å)	3.4	6.4
FSC threshold	0.143	0.143
Model resolution range (Å)	N/A	N/A

Map sharpening *B* factor (Å^2)	-88.99	-129.74
Model composition		
Non-hydrogen atoms	9,928	9,928
Protein residues	1,180	1,180
Nucleotide residues	53	53
Ligands	1	1
R.m.s. deviations		
Bond lengths (Å)	0.012	0.013
Bond angles (°)	1.125	1.344
Validation		
MolProbity score	2.02	2.03
Clashscore	8.07	8.34
Poor rotamers (%)	1.30	1.30
Ramachandran plot		
Favored (%)	91.88	91.88
Allowed (%)	8.12	8.12
Disallowed (%)	0.00	0.00
Model vs Data		
CC (mask)	0.70	0.54
CC (box)	0.75	0.76
CC (peaks)	0.64	0.45
CC (volume)	0.63	0.55

Supplementary Table 2. Quantitation of the median DNA synthesis by Pol κ mutants

For Panel C

Lane	2	3	4	5	6	7	8	9	10	11	12	13
dNRFC		+	+	-	+	+	-	+	+	-	+	+
Ub-PCNA	-	-	+	-	-	+	-	-	+	-	-	+
wt-PCNA	-	+	-	-	+	-	-	+	-	-	+	_
DR-Pol k	-	-	-	-	-	-	-	-	-	+	+	+
QRSI-Pol k	-	-	-	-	-	-	+	+	+	-	-	-
IFL-Pol k	-	-	-	+	+	+	-	-	-	-	-	-
Pol k	+	+	+	-	-	-	-	-	-	-	-	-
$N_{1/2}$ (nt)	11.0	16.6	17.3	9.4	11.0	14.8	7.9	9.0	14.4	10.5	18.8	18.1
t (s)	40.0											
$k_{1/2}$ (nt/s)	0.27	0.42	0.43	0.24	0.28	0.37	0.20	0.23	0.36	0.26	0.47	0.45
$k_{1/2}$-folds stimulation (X)	1.0	1.5	1.6	0.9	1.0	1.3	0.7	0.8	1.3	1.0	1.7	1.7

For Panel D

Lane	2	3	4	5	6	7	8	9	10	11	12	13
dNRFC	-	+	+	-	+	+	-	+	+	-	+	+
Ub-PCNA	-	-	+	-	-	+	-	-	+	-	-	+
wt-PCNA	-	+	-	-	+	-	-	+	-	-	+	_
IFLDD-Pol k	-	-	-	-	-	-	-	-	-	+	+	+
IFLD799-Pol k	-	-	-	-	-	-	+	+	+	-	-	-
IFLD644-Pol k	-	-	-	+	+	+	-	-	-	-	-	-
IFL-Pol k	+	+	+	-	-	-	-	-	-	-	-	-
$N_{1/2}$ (nt)	5.4	6.4	11.6	6.1	6.7	8.0	6.8	6.1	7.5	5.8	6.0	7.0
t (s)	40.0											
$k_{1/2}$ (nt/s)	0.14	0.16	0.29	0.15	0.17	0.20	0.17	0.15	0.19	0.14	0.15	0.17
$k_{1/2}$-folds stimulation (X)	1.0	1.2	2.1	1.1	1.2	1.5	1.3	1.1	1.4	1.1	1.1	1.3

Table legend: Quantitation of the median DNA synthesis from the SDS-PAGE gels shown in main text Fig. 5 panels C and D, respectively. The $N_{1/2}$ values for each lane were obtained from the fittings presented in main text Fig. 5e as described in the corresponding figure legend and in the Materials and Methods section. Each $N_{1/2}$ value was converted to a median DNA synthesis rate ($k_{1/2}$) by dividing the $N_{1/2}$ value by the indicated experimental total extension time. For the table corresponding to the gel illustrated in main text Fig. 5C, the $k_{1/2}$ values are compared, as fold difference, with the value corresponding to WT Pol κ alone. For the table corresponding to the gel illustrated in main text Fig. 5D, the $k_{1/2}$ values are compared, as fold difference, with the value corresponding to IFL-Pol κ alone.

References

1. Zi Tan, Y. *et al.* Addressing preferred specimen orientation in single-particle cryo-EM through tilting. *Nat. Methods* **14**, 793–796 (2017).

2. Lone, S. *et al.* Human DNA Polymerase κ Encircles DNA: Implications for Mismatch Extension and Lesion Bypass. *Mol. Cell* **25**, 601–614 (2007).

3. Lancey, C. *et al.* Structure of the processive human Pol δ holoenzyme. *Nat. Commun.* **11**, 1109 (2020).
