# Supplementary material for: Cryo-EM structure of human Pol κ bound to DNA and mono-ubiquitylated PCNA
Source: Nat Commun. 2021 Oct 19;12:6095. doi: 10.1038/s41467-021-26251-6 (PMC8526622; doi:10.1038/s41467-021-26251-6)
Supplement: Supplementary file 3 — Description of Additional Supplementary Files [file 41467_2021_26251_MOESM3_ESM.pdf]

Description of Additional Supplementary Files

File Name: Supplementary Movie 1
Description: Apo1 MD trajectory of Polκ-PCNA complex. Colour code: Polκ core in bright orange; Polκ PAD in green; PAD C-terminus in red; PCNA in sky blue.

File Name: Supplementary Movie 2
Description: Apo1b MD trajectory of Polκ-PCNA complex. Colour code: Polκ core in bright orange; Polκ PAD in green; PAD C-terminus in red; PCNA in sky blue.

File Name: Supplementary Movie 3
Description: Apo2 MD trajectory of Polκ-PCNA complex. Colour code: Polκ core in bright orange; Polκ PAD in green; PAD C-terminus in red; PCNA in sky blue.

File Name: Supplementary Movie 4
Description: Apo2b MD trajectory of Polκ-PCNA complex. Colour code: Polκ core in bright orange; Polκ PAD in green; PAD C-terminus in red; PCNA in sky blue.
