# Supplementary material for: Cryo-EM structure of human Pol κ bound to DNA and mono-ubiquitylated PCNA
Source: Nat Commun. 2021 Oct 19;12:6095. doi: 10.1038/s41467-021-26251-6 (PMC8526622; doi:10.1038/s41467-021-26251-6)
Supplement: Supplementary file 8 — Reporting Summary [file 41467_2021_26251_MOESM8_ESM.pdf]

nature research

Corresponding author(s): Alfredo De Biasio and Samir H. Hamdan

Last updated by author(s): September 07, 2021

Reporting Summary

Nature Research wishes to improve the reproducibility of the work that we publish. This form provides structure for consistency and transparency in reporting. For further information on Nature Research policies, see Authors & Referees and the Editorial Policy Checklist .

Statistics

For all statistical analyses, confirm that the following items are present in the figure legend, table legend, main text, or Methods section.

n/a	Confirmed	
☐	☒	The exact sample size (*n*) for each experimental group/condition, given as a discrete number and unit of measurement
☒	☐	A statement on whether measurements were taken from distinct samples or whether the same sample was measured repeatedly
☒	☐	The statistical test(s) used AND whether they are one- or two-sided<br>*Only common tests should be described solely by name; describe more complex techniques in the Methods section.*
☒	☐	A description of all covariates tested
☒	☐	A description of any assumptions or corrections, such as tests of normality and adjustment for multiple comparisons
☐	☒	A full description of the statistical parameters including central tendency (e.g. means) or other basic estimates (e.g. regression coefficient) AND variation (e.g. standard deviation) or associated estimates of uncertainty (e.g. confidence intervals)
☒	☐	For null hypothesis testing, the test statistic (e.g. *F*, *t*, *r*) with confidence intervals, effect sizes, degrees of freedom and *P* value noted<br>*Give P values as exact values whenever suitable.*
☒	☐	For Bayesian analysis, information on the choice of priors and Markov chain Monte Carlo settings
☒	☐	For hierarchical and complex designs, identification of the appropriate level for tests and full reporting of outcomes
☒	☐	Estimates of effect sizes (e.g. Cohen's *d*, Pearson's *r*), indicating how they were calculated

Our web collection on statistics for biologists contains articles on many of the points above.

Software and code

Policy information about availability of computer code

Data collection	Cryo-EM data collection was performed using Thermofisher Scientific EPU v1.9 and v2.3 software
Data analysis	Cryo-EM data analysis was performed using Relion 3.1-beta software; Gel data were analysed with GelAnalyser v19.1; NMR data were analysed with Topspin and NMRFAM-Sparky; MD models were analysed with Modeller v10.1, DES-Amber, Gromacs 2019.4, MDTraJ, Matplotlib, ENCORE, VMD, and Pymol; Structural models were analysed using Pymol, HHPred, Coot v0.9.6, USFC Chimera and Phenix v1.19.2

For manuscripts utilizing custom algorithms or software that are central to the research but not yet described in published literature, software must be made available to editors/reviewers. We strongly encourage code deposition in a community repository (e.g. GitHub). See the Nature Research guidelines for submitting code & software for further information.

Data

Policy information about availability of data

All manuscripts must include a data availability statement. This statement should provide the following information, where applicable:

- Accession codes, unique identifiers, or web links for publicly available datasets
- A list of figures that have associated raw data
- A description of any restrictions on data availability

The maps of the Pol kappa-DNA-PCNA complex and Pol kappa-DNA-UbPCNA complex have been deposited in the EMBD with accession codes EMD-12601, EMD-12602, and the atomic models in the Protein Data Bank under accession codes PDB 7NV0 and 7NV1. Additional atomic models used in this study are: Pol k catalytic domain bound to DNA (PDB ID 2OH2); apo Pol k (PDB ID 1T94); PCNA homotrimer (from PDB ID 6TNZ); Rad18-UBZ/ubiquitin complex (PDB ID 2MRE); Pol delta holoenzyme (PDB ID 6TNY and 6S1O); mono-ubiquitylated PCNA (PDB ID 3L0W and 3TBL).

The authors declare no competing financial interests. Correspondence and requests for materials should be addressed to S.M.H. (samir.hamdan@kaust.edu.sa) or A.D.B. (adb43@leicester.ac.uk).

Field-specific reporting

Please select the one below that is the best fit for your research. If you are not sure, read the appropriate sections before making your selection.

☒ Life sciences ☐ Behavioural & social sciences ☐ Ecological, evolutionary & environmental sciences

For a reference copy of the document with all sections, see nature.com/documents/nr-reporting-summary-flat.pdf

Life sciences study design

All studies must disclose on these points even when the disclosure is negative.

Sample size	The sample size was determined based on the target near atomic resolution in the cryo-EM maps.
Data exclusions	No data were excluded from the bulk DNA replication assays; micrographs containing limited resolution information were discarded. Particles contributing to low-resolution 2D and 3D averages were also discarded.
Replication	Bulk DNA replication assays were repeated at least two times; cryo-EM experiments were highly reproducible: the map of PolK-DNA-PCNA complex was obtained from two independently acquired datasets.
Randomization	Randomization is not relevant to this study as it only includes cryo-EM, NMR and bulk DNA assay data.
Blinding	Blinding is not relevant to this study as it only includes cryo-EM, NMR and bulk DNA assay data.

Reporting for specific materials, systems and methods

We require information from authors about some types of materials, experimental systems and methods used in many studies. Here, indicate whether each material, system or method listed is relevant to your study. If you are not sure if a list item applies to your research, read the appropriate section before selecting a response.

Materials & experimental systems

n/a	Involved in the study	
☒	☐	Antibodies
☒	☐	Eukaryotic cell lines
☒	☐	Palaeontology
☒	☐	Animals and other organisms
☒	☐	Human research participants
☒	☐	Clinical data

Methods

n/a	Involved in the study	
☒	☐	ChIP-seq
☒	☐	Flow cytometry
☒	☐	MRI-based neuroimaging
